# Novel Carbon/PEDOT/PSS-Based Screen-Printed Biosensors for Acetylcholine Neurotransmitter and Acetylcholinesterase Detection in Human Serum

**DOI:** 10.3390/molecules24081539

**Published:** 2019-04-18

**Authors:** Nashwa H. Ashmawy, Abdulrahman A. Almehizia, Teraze A. Youssef, Abd El-Galil E. Amr, Mohamed A. Al-Omar, Ayman H. Kamel

**Affiliations:** 1 Chemistry Department, Faculty of Science, Ain Shams University, P.O. Cairo 11566, Egypt; nashwastar20@yahoo.com (N.H.A.); trease_albert@yahoo.com (T.A.Y.); 2 Pharmaceutical Chemistry Department, College of Pharmacy, King Saud University, Riyadh 11451, Saudi Arabia; mehizia@ksu.edu.sa (A.A.A.); malomar1@ksu.edu.sa (M.A.A.-O.); 3 Pharmaceutical Chemistry Department, Drug Exploration & Development Chair (DEDC), College of Pharmacy, King Saud University, Riyadh 11451, Saudi Arabia; 4 Applied Organic Chemistry Department, National Research Centre, Dokki, Giza 12622, Egypt

**Keywords:** solid-contact/ISEs, PEDOT/PSS, acetylcholine, biosensors, acetylcholinesterase

## Abstract

New reliable and robust potentiometric ion-selective electrodes were fabricated using poly(3,4-ethylenedioxythiophene)/poly(styrenesulfonate) (PEDOT/PSS) as the solid contact between the sensing membrane and electrical substrate for an acetylcholine (ACh) bioassay. A film of PEDOT/PSS was deposited on a solid carbon screen-printed platform made from ceramic substrate. The selective materials used in the ion-selective electrode (ISE) sensor membrane were acetylcholinium tetraphenylborate (ACh/TPB/PEDOT/PSS-ISE) (sensor I) and triacetyl-β-cyclodextrin (β-CD/PEDOT/PSS-ISE) (sensor II). The sensors revealed clear enhanced Nernstian response with a cationic slope 56.4 ± 0.6 and 55.3 ± 1.1 mV/decade toward (ACh^+^) ions over the dynamic linear range 1.0 × 10^−6^–1 × 10^−3^ and 2.0 × 10^−6^–1.0 × 10^−3^ M at pH 5 with limits of detection 2.0 × 10^−7^ and 3.2 × 10^−7^ M for sensors I and II, respectively. The selectivity behavior of both sensors was also tested and the sensors showed a significant high selectivity toward ACh^+^ over different common organic and inorganic cations. The stability of the potential response for the solid-contact (SC)/ISEs was evaluated using a chronopotentiometric method and compared with that of electrodes prepared without adding the solid-contact material (PEDOT/PSS). Enhanced accuracy, excellent repeatability, good reproducibility, potential stability, and high selectivity and sensitivity were introduced by these cost-effective sensors. The sensors were also used to measure the activity of acetylcholinesterase (AChE). A linear plot between the initial rate of the hydrolysis of ACh^+^ substrate and enzyme activity held 5.0 × 10^−3^–5.2 IU L^−1^ of AChE enzyme. Application to acetylcholine determination in human serum was done and the results were compared with the standard colorimetric method.

## 1. Introduction

Neurotransmitters are classified as important endogenous chemical messengers. These messengers transmit and enhance specific signals between neurons and other cells. They have an important role in behavior and cognition functions in the brain. In addition, they can play an important role in muscle tone and heart rate adjustment. Also, they are responsible for regulation of learning, sleeping, memory, consciousness, mood, and appetite in human bodies [1,2]. Acetylcholine (ACh) can be considered as one of the oldest neurotransmitters in the animal kingdom in both peripheral and central nervous systems [2]. It binds to its specific receptors to regulate muscle contraction in the peripheral nervous system. On the other hand, it plays an essential role in the central nervous system in the processes related to behavioral activities. In neurons, ACh is prepared from choline using choline acetyltransferase (ChAT) and acetylcoenzyme A [1,2,3]. The level of ACh in human blood is ~0.52 µM in males and ~0.47 µM in females [4]. ACh also has a therapeutic utility as an intraocular irrigating fluid [5]. On the other hand, a lack of ACh can cause disturbance in the transmission of nerve impulses, paralysis, and death [6].

From all of the above, the level of ACh in human blood is very critical and it is very important to find an analytical tool to trace its level in the human body. The development of reliable and robust analytical devices for fast and sensitive assessment of ACh was a great challenging target for many years. ACh is not ultraviolet (UV)-absorbing, has no fluorescence signal, and is not derivatizable. Thus, tracing of ACh is a challenging analytical problem. Therefore, only tedious procedures such as bioassays [7], methods based on radiochemical analysis [8], enzymatic-based liquid chromatography (LC) [9,10,11,12], and mass spectrometric detection-based LC [13,14,15,16] are often used. In addition, theses reported methods have several disadvantages, such as a long analysis time, high cost analysis, and the requirement of highly skilled people specialized with laboratory facilities. 

Electroanalytical techniques provide many advantages such as simple equipment used for analysis, ease of use, and a short time taken for analysis. Uni-, bi-, and tri-enzyme/mediator biosensors including chemiluminometric [17], amperometric [18,19,20], conductometric [21], and voltammetric [22] methods are conducted for ACh monitoring. On the other hand, all these mentioned methods suffer from a long analysis time and the need for specialized personnel with laboratory facilities. 

In comparison with different techniques, potentiometric ion-selective electrodes (ISEs) have some unique performance characteristics, such as ease of miniaturization, ease of handling, portability, and low cost [23,24,25,26]. Recently, solid-state potentiometric ion-selective sensors revealed a tremendous ability to analyze biological, biomedical, and environmental samples [27,28,29]. The configuration of all-solid-state ISEs seems suitable for miniaturized sensor fabrication characterized with good analytical characteristics to detect different ions in different matrices. 

As cited in the literature, few potentiometric ion-selective electrodes (ISEs) were developed [30,31,32,33,34,35,36]. Some of them include ion-pair complexes of acetylcholine as electro-active materials. These revealed limited linear range and a long response time of analysis [30,31,32]. The others include macrocycle carriers such as β-cyclodextrin and molecularly imprinted polymers [33,35,36]. It is interesting to compare the selectivity and working of the proposed ACh electrode along with those reported before (Table 1). Traditional potentiometric ISE configurations containing inner filling solution presented good performance characteristics for benchtop ion sensing. However, this configuration fails with small-volume samples. These types of samples need to be addressed along with the requirements of miniaturization. Considering in vivo biomedical analysis, the use of liquid-contact ion-selective electrodes (ISEs) has many drawbacks. For example, the inner filling solution is subjected to evaporation, as well as changes in temperature and pressure of the sample. In addition, osmotic pressure can be caused due to differences in the ionic strength of the sample and the inner filling solution. This arisen osmotic pressure can produce a net liquid transport from/to the inner filling solution and lead to volume changes. This can provoke delamination of the ISM [37]. As an example of solid-contact electrodes, screen-printed electrodes were shown to be a good technique for sensor miniaturization, as they are simple, cheap, and easy to be fabricated for mass production. These good features of screen-printed electrodes make such electrode types promising for the detection of organic species.

Herein, we present novel robust, reliable potentiometric solid-contact ISEs for trace determination of acetylcholine. A poly(3,4-ethylenedioxythiophene)/poly(styrenesulfonate) (PEDOT/PSS) film was used as the solid contact. The sensors were introduced for the determination of ACh under static and hydrodynamic modes of operation. The proposed sensors were also applied for simple, sensitive, and rapid monitoring of acetylcholinesterase (AChE) enzyme activities. The method for enzyme monitoring was based on the reaction of the enzyme with ACh^+^ substrate, while monitoring the decrease of ACh^+^ concentration using the proposed ACh sensor. 

## 2. Results and Discussion

### 2.1. Sensors Performance Characteristics

**Figure 1:**
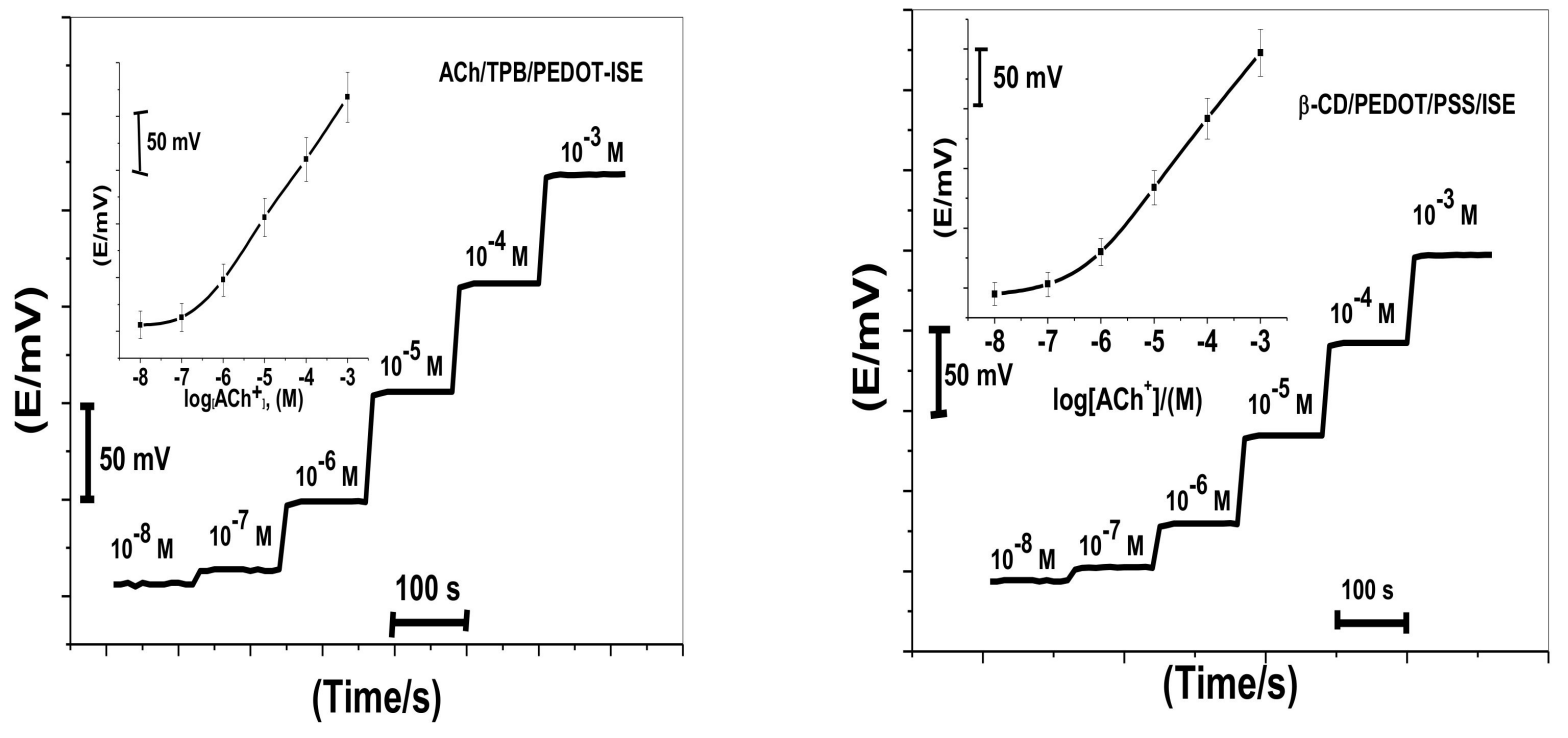
Potentiometric responses of the proposed sensors.

Acetylcholine reacts with sodium tetraphenylborate forming an acetylcholinium-tetraphenylborate (ACh/TPB) ion-associated complex in a stoichiometric ratio of 1:1. The resulting precipitate was purified, isolated, and dried. Two polymeric potentiometric acetylcholine sensors were based on the use of ACh/TPB (ACh/TPB/PEDOT/PSS-ISE) and tri acetyl-β-cyclodextrin (β-CD/PEDOT/PSS-ISE) in a plasticized poly(vinyl chloride) (PVC) matrix. The polymeric membrane composition of the two sensors was 63.1 wt.% plasticizer, 32.4 wt.% PVC, 1.5 wt.% tetradodecylammonium tetrakis (4-chlorophenyl) borate (ETH 500), and 3.0 wt.% ionophore. All calibration plots for the proposed electrodes are shown in Figure 1. The obtained results from triplicate studies revealed a near-Nernstian slope of 56.4 ± 0.6 (*R^2^* = 0.999) and 55.3 ± 1.1 (*R^2^* = 0.998) mV/decade, with detection limits of 2.00 × 10^−7^ and 3.20 × 10^−7^ M, for sensors I and II, respectively. The performance response characteristics are tabulated in Table 2.

The influence of pH on the proposed sensors was also investigated. Two different concentrations of ACh^+^ (10^−4^–10^−3^ M) were chosen for this test over a pH range of 2 to 10. Sensors I and II revealed stable and unaffected potentials at the presented concentrations by pH changes over the working pH ranges 3–10 and 4.5–10, respectively. At pH > 10, the potentials sharply decreased due to interference from hydroxide ions. At pH < 3, the sensor responses were severely influenced by H_3_O^+^. A 10^−2^ M concentration of phosphate-buffered saline (PBS) at pH 7 was chosen for all subsequent measurements.

The closeness of agreement between mutually independent repetitive test results obtained with a 10 µg/mL internal quality control ACh sample was measured using the proposed sensors and the same reagents during short intervals of time within one working day (within-day reproducibility), and a significantly small variation (±0.5%) from the final mV readings was noticed. Reproducibility of the results (day-to-day response variations) was also tested by measuring a 10 µg/L internal quality control ACh sample on five consecutive days using different batches of the reagents and daily recalibration. A small variation of the results compared to those obtained for repeatability experiments was obtained. Calculation of the relative standard deviation was found to be 1.7% and 2.2% for ACh/TPB/PEDOT/PSS-ISE and β-CD/PEDOT/PSS-ISE, respectively. These data indicate the good response stability of the proposed solid-contact (SC)/ISEs. After repeating the calibration of the sensors for at least 12 weeks, the long-term potential stability was tested. After six weeks of daily use, the detection limit increased to 7.0 × 10^−5^ M for both sensors and the sensitivity was found to decline. Considering the disposable nature of these types of sensors, the issue of decreasing sensitivity after six weeks was not taken as a big problem.

### 2.2. Sensor Selectivity

Selectivity behavior of the proposed sensors toward acetylcholine was tested over different common ions. The selectivity coefficients (*K_ACh,J_*) were evaluated using the separate solution method (SSM) modified by Bakker [38]. Table 3 presents the potentiometric selectivity coefficient values of all sensors. From the results shown in Table 3, the selectivity of the sensor based on β-CD (β-CD/PEDOT/PSS-ISE) toward ACh^+^ ions over choline, methylamine, urea, dimethylamine, Na^+^, Ca^2+^, and Mg^2+^ ions was much better than the sensor based on ACh/TPB (ACh/TPB/PEDOT/PSS-ISE). This can be explained on the basis of the high affinity of tri acetyl-β-CD toward complexation of acetylcholine as compared to other nitrogenous compounds. On the other hand, the sensor based on ACh/TPB/PEDOT/PSS-ISE (sensor I) revealed better selectivity toward ACh^+^ over K^+^, histidine, ethylene diamine, hexamine, hydroxylamine, ephedrine, codeine, morphine, and alanine than β-CD/PEDOT/PSS-ISE.

### 2.3. Effect of the PEDOT/PSS Solid-Contact Layer

**Figure 2:**
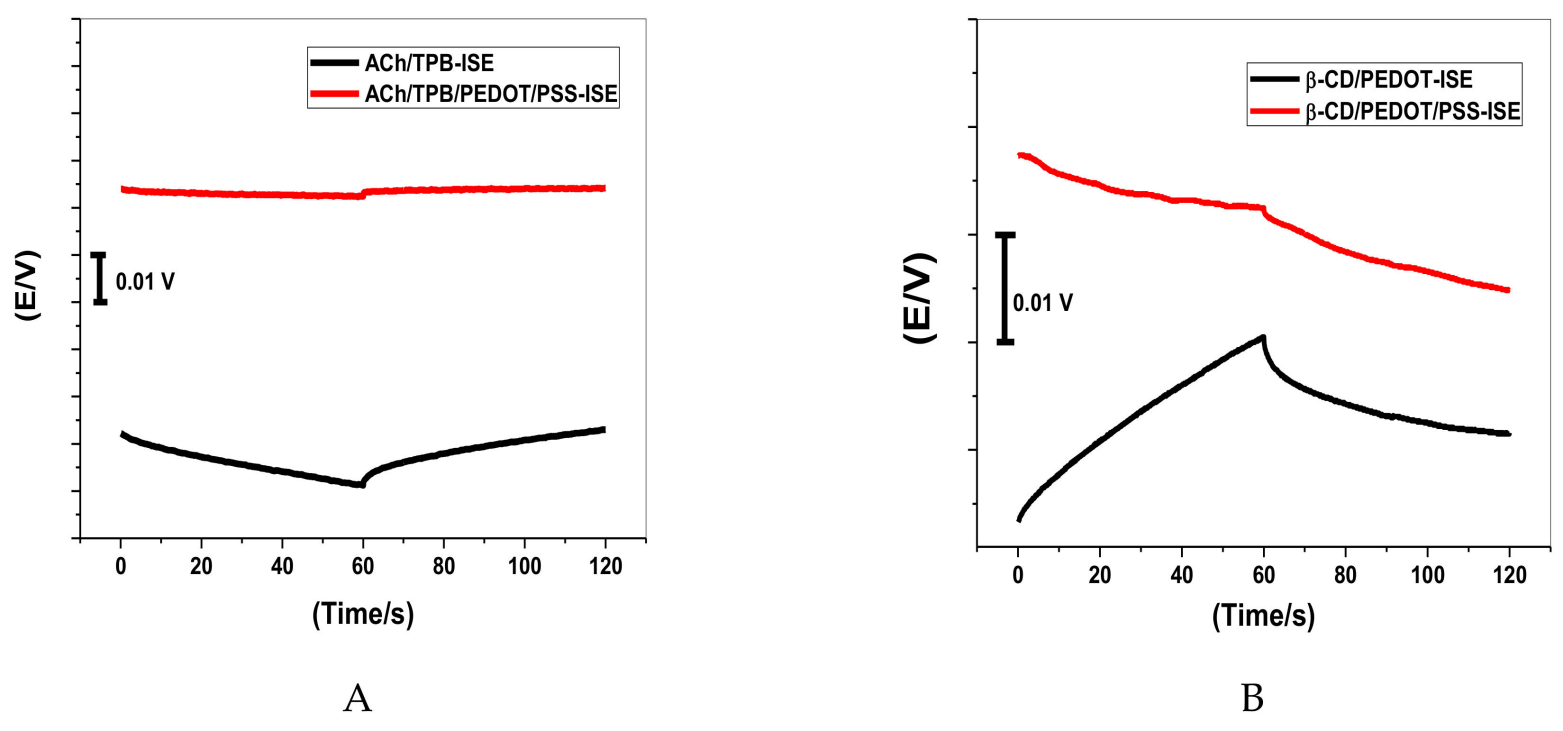
Chronopotentiograms (applied current: +1 nA for 60 s and −1 nA for 60 s) for all-solid-state acetylcholine (ACh^+^) ion-selective electrode (ISE) based on (**A**) tetraphenylborate (TPB) and (**B**) β-cyclodextrin (β-CD).

Short-term potential stability was studied using chronopotentiometry [39]. As shown in Figure 2, the typical chronopotentiograms of ACh/PEDOT/PSS-ISEs and β-CD/PEDOT/PSS-ISE, in addition to that of ACh/TPB-ISE and β-CD-ISE, are presented for comparison. The slope (Δ*E*/Δ*t*) of the *E–t* curve at longer times gives a direct measure of the potential stability of the ACh+-ISEs. The potential drifts were 6.5 μV/s and 52.0 μV/s for ACh/TPB/PEDOT/PSS-ISE and β-CD/PEDOT/PSS-ISE, respectively, which were much lower than those of ACh/TPB-ISE and β-CD-ISE (117.0 μV/s and 90.2 μV/s, respectively). From the data presented above, it was found that the potential stability of the proposed sensors was greatly improved by the introduction of PEDOT/PSS directly into the polymeric membrane. The capacitances were estimated to be 153.1 μF and 19.1 μF for ACh/TPB/PEDOT/PSS-ISE and β-CD/PEDOT/PSS-ISE, respectively. The capacitances of ACh/TPB-ISE and β-CD-ISE were 8.54 μV/s and 11.0 μV/s, respectively. From these values, we can confirm the relationship between potential stability (Δ*E*/Δ*t*) or the capacitance (*C*) of ISEs and the presence of PEDOT/PSS as a solid-contact material between the membrane and the electrical substrate in the ISEs.

### 2.4. Water-Layer Effect

**Figure 3:**
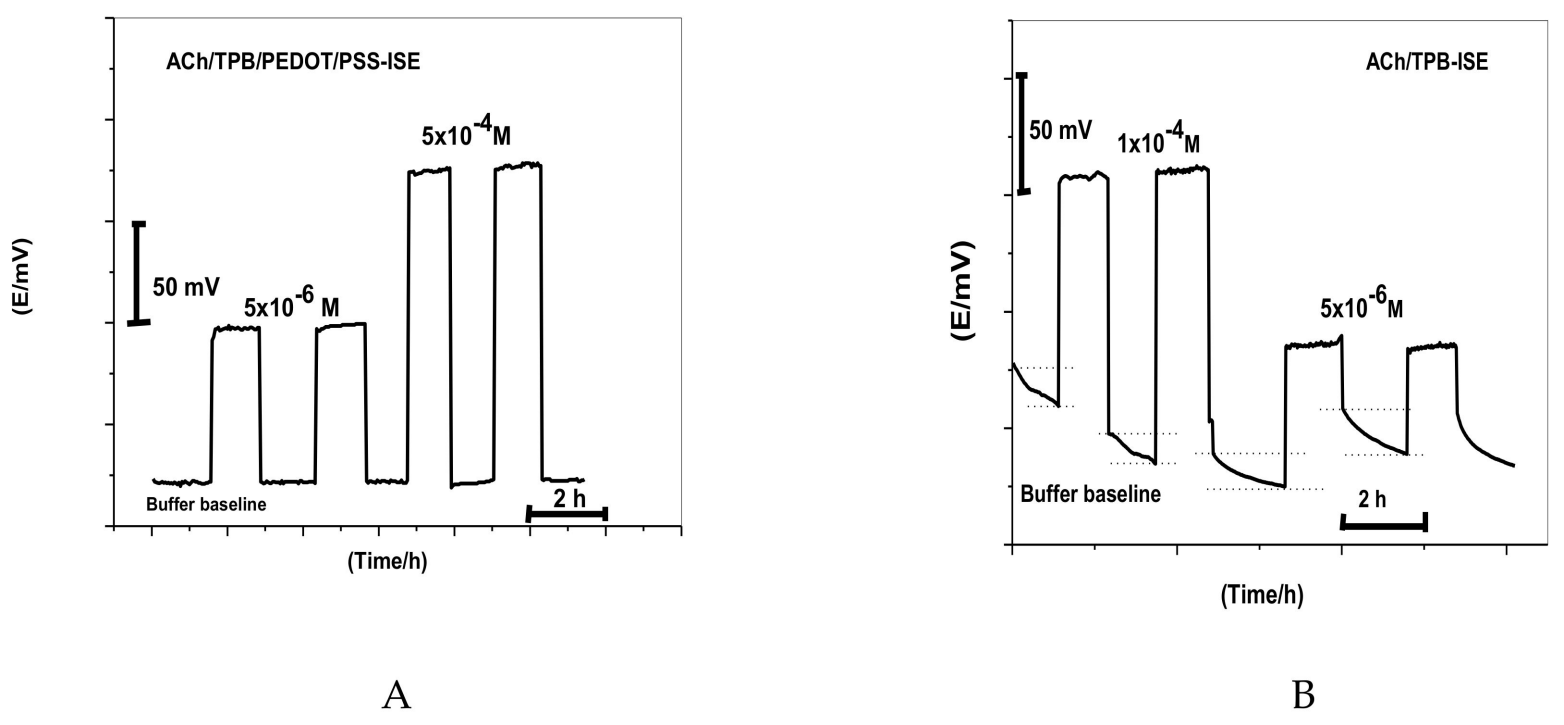
Water-layer tests for the acetylcholine ISE with and without poly(3,4-ethylenedioxythiophene)/poly(styrenesulfonate) (PEDOT/PSS) solid-contact material for (**A**) ACh/TPB/PEDOT/PSS-ISE and (**B**) ACh/TPB-ISE.

The water film formed between the ion-sensing membrane and electron conductor interface can act as localized microscopic pools of water [40]. It has a great influence on the potential stability and lifetime of the ISE. Thus, a test for water-layer absence was carried out for ACh/TPB/PEDOT/PSS-ISE. As shown in Figure 3, the proposed sensor was firstly conditioned in 10^−3^ M CaCl_2_ solution and the potential was then recorded for 1.0 h. After that, the solution of CaCl_2_ was replaced with 1.0 × 10^−4^ and 5 × 10^−6^ M ACh^+^ solution, and the potential was recorded for another 1.0 h. After replacing CaCl_2_ solution with ACh^+^ ion solution, the stable potential response for nearly 1.0 h showed complete absence of the undesirable water layer. This can be explained on the basis of the hydrophobic nature of PEDOT/PSS in the polymeric membrane. A similar ACh^+^ selective membrane composition without PEDOT/PSS was also tested for comparison. A great potential drift was noticed in the absence of the PEDOT/PSS layer as a solid-contact material. This confirms the hydrophobic nature of this material and the absence of water-layer formation.

### 2.5. Hydrodynamic Assessment of ACh^+^

**Figure 4:**
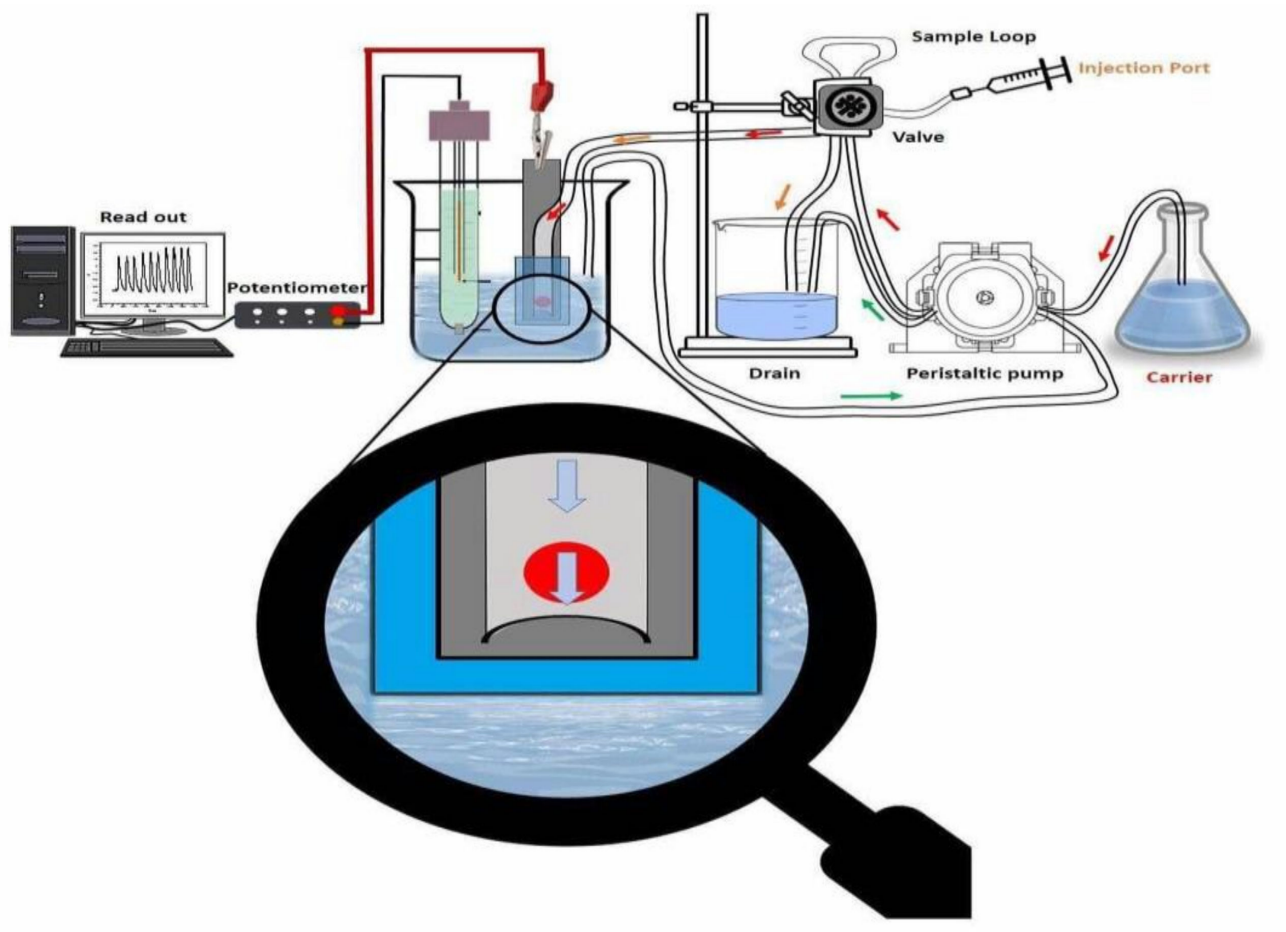
Flow injection manifold for ACh^+^ quantification. Conditions: phosphate-buffered saline (PBS) (0.01 M, pH 7.0) carrier solution; 100 μL was the injected sample volume with a 4.0 mL/min flow rate.

**Figure 5:**
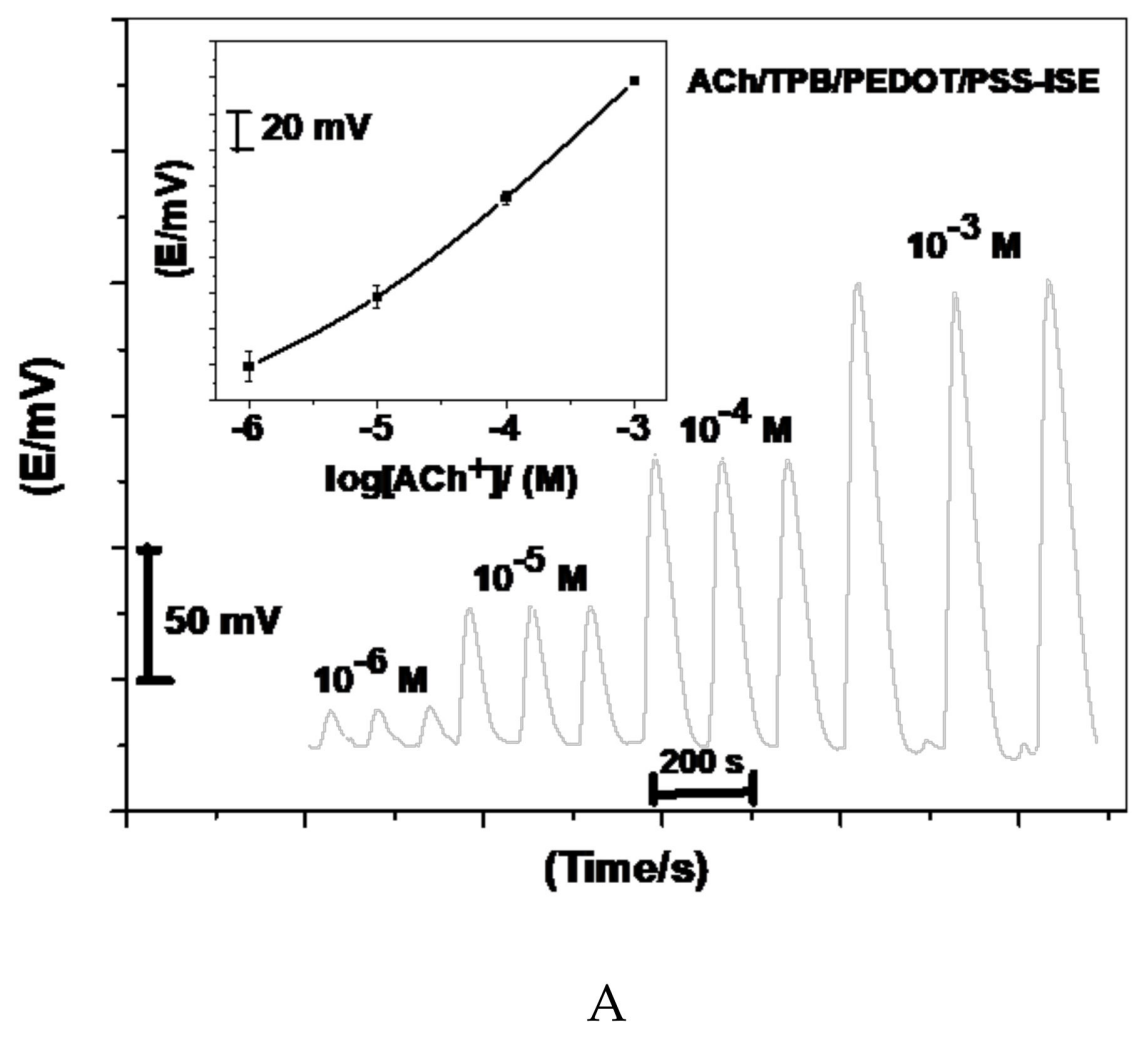

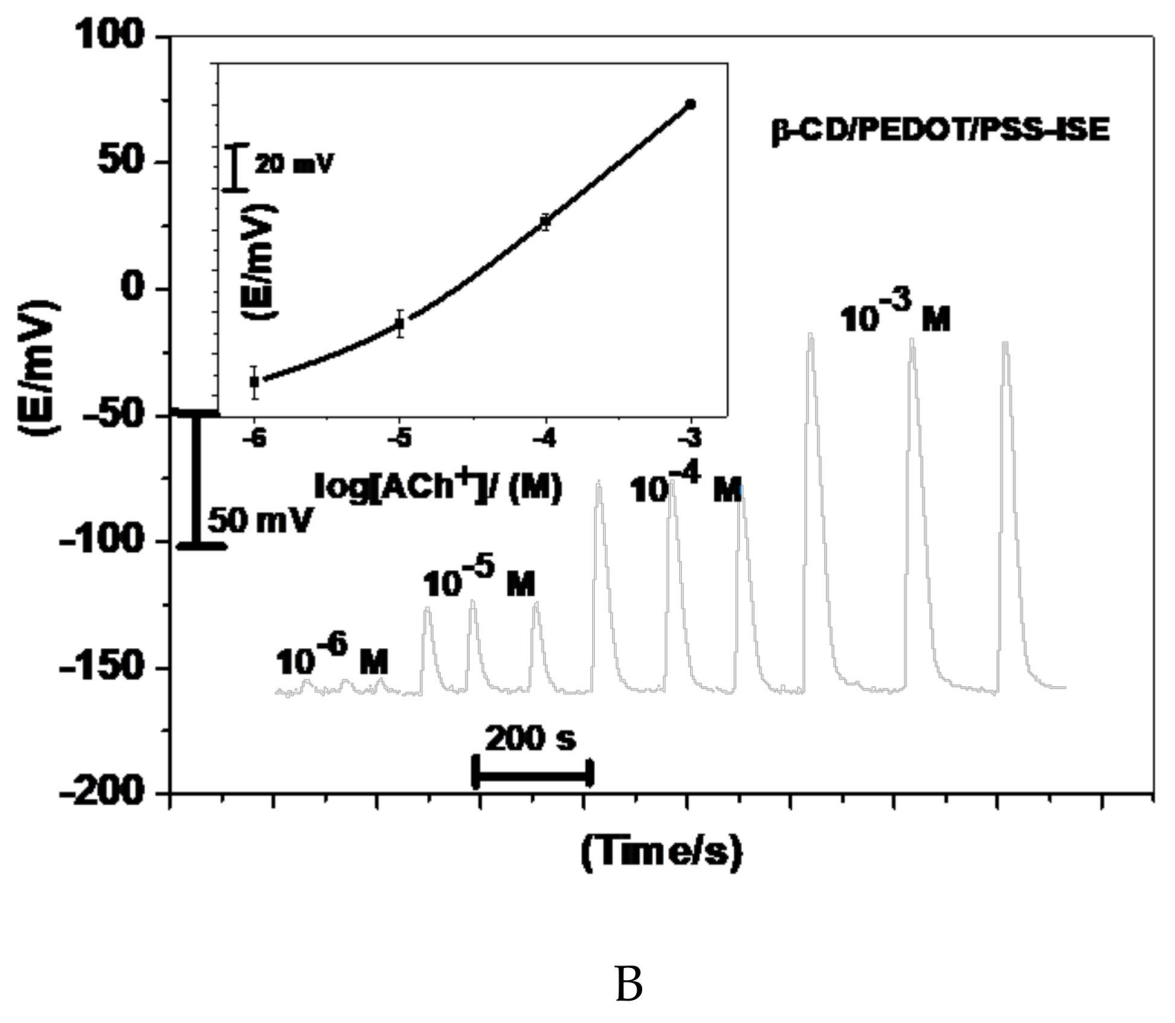
Flow injection signals for the evaluation of ACh^+^ under the optimized flow injection conditions using: (**A**) ACh/TPB/PEDOT/PSS-ISE and (**B**) β-CD/PEDOT/PSS-ISE.

The flow injection manifold of the system is shown in Figure 4. The flow cell used was prepared to afford a small sensor size. This design was used to avoid sample dispersion and to obtain a short recovery time for the potential response. Planar-type detectors (i.e., screen-printed electrodes) containing ACh/TPB- and β-CD-based membrane sensors were prepared and characterized by measuring ACh^+^ ions under flow-through operation. The recorded potential signals of the sensors are presented in Figure 5. The sensors revealed a Nernstian response slope of 60.1 ± 1.1 and 52.7 ± 0.8 mV/decade over the linear range 1.0 × 10^−5^ to 1.0 × 10^−3^ M and 2.0 × 10^−5^ to 1.0 × 10^−3^ M with detection limits of 2.5 × 10^−6^ and 3.9 × 10^−6^ M for ACh/TPB/PEDOT/PSS-ISE and β-CD/PEDOT/PSS-ISE, respectively. A value of 4 mL/min was recommended as an optimized flow rate for measuring. All potentiometric characteristics are summarized in Table 4. The relative standard deviation of the transient flow signals was ± 2.1% and ± 1.7% over the concentration range 1.0 × 10^−6^ to 1.0 × 10^−2^ M ACh^+^ ions for the proposed sensors, respectively.

### 2.6. Acetylcholine Assay in Human Serum

The usefulness of the proposed method was tested by determining ACh in collected human serum samples. For comparison with the present potentiometric procedure, the samples were also analyzed using a standard commercial spectrophotometric kit (No. ab65345, Abcam, Boston, MA, USA) at 25 °C. In this assay protocol, free choline is oxidized by choline oxidase to betaine and H_2_O_2_. The hydrogen peroxide is then detected with a highly specific colorimetric probe. Horseradish peroxidase is used as a catalyst for this reaction. The reaction products generate a color measure at λ = 540 to 570 nm. The results obtained from the standard and proposed methods are presented in Table 5. An *F*-test showed that there was no significant difference between means and variances of both the spectrophotometric and potentiometric sets of results.

In addition, four different sensor assemblies with two different instruments on different days were used for repetitive determination of different sample sizes of ACh. Repeatability (within-day) and reproducibility (between-day) measurements showed potential variation in the range of 2–3 mV. These results revealed that the influence of these parameters was within the specified tolerance and the variations are considered within the method’s robustness range.

### 2.7. Kinetic Monitoring of Acetylcholinesterase Activity

**Scheme 1:**
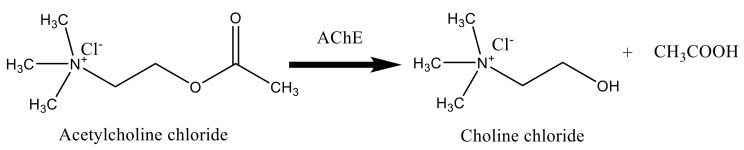
Hydrolysis of AChCl by acetylcholinesterase (AChE).

Termination of the transmitted pulses in the cholinergic synapses is done via the fast hydrolysis of acetylcholine (ACh) into choline (Ch) and acetic acid as shown in Scheme 1 [41].

In the enzymatic reaction, the reaction rate when the enzyme is saturated with substrate can be considered as the maximum rate of reaction (*V_max_*), and *K_m_* is the concentration of substrate which permits the enzyme to achieve half *V_max_*. The values of *K_m_* and *V_max_* of the enzymatic reaction were estimated using the proposed Ach sensor.

**Figure 6:**
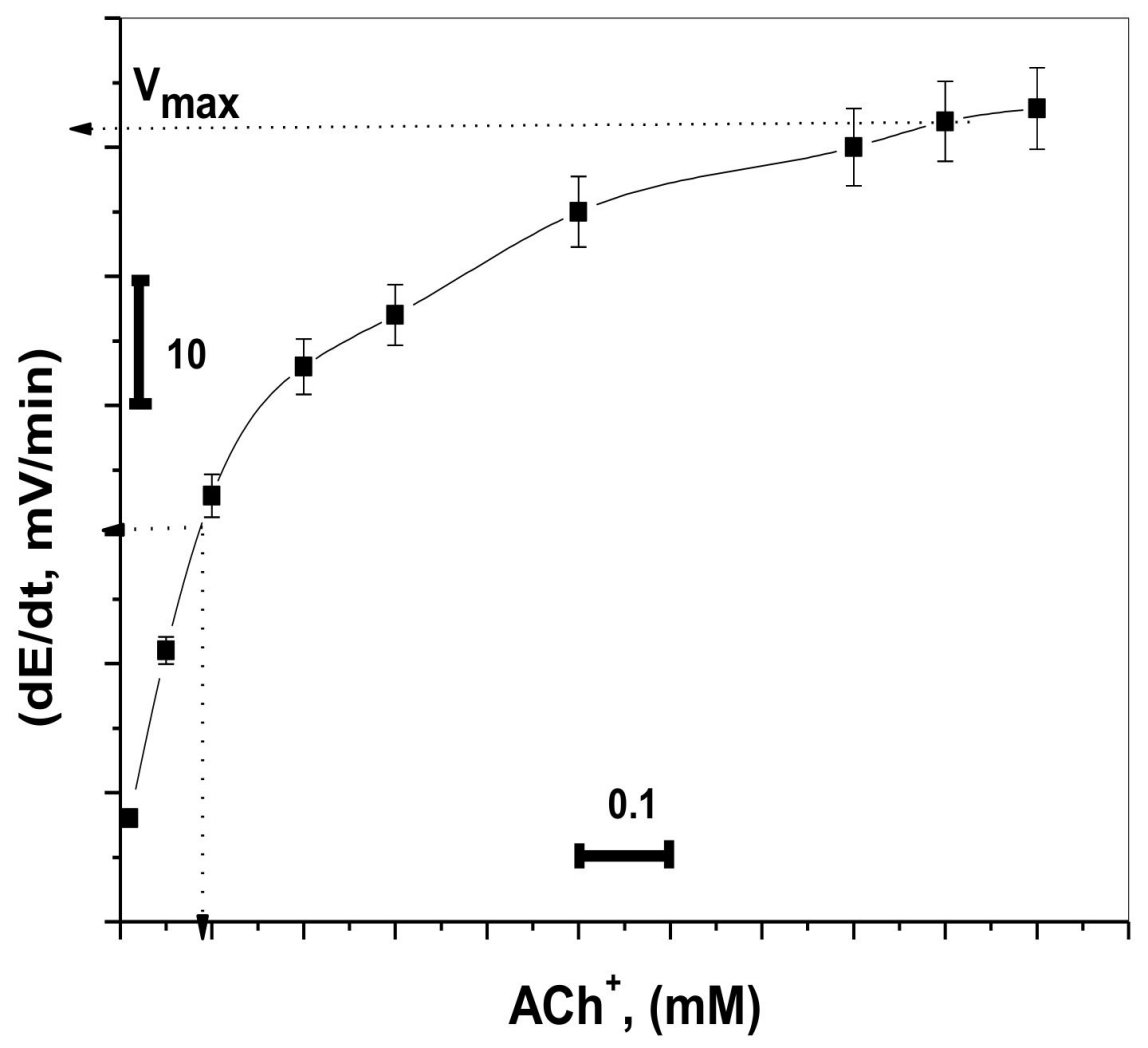
Initial reaction rates obtained by the proposed sensor fitted to the Michaelis–Menten equation (acetylcholinesterase (AchE) activity = 0.5 IU/L).

The potential change was recorded for different concentrations of ACh^+^ (1.0 × 10^−6^ to 1.0 × 10^−3^ M) using fixed enzyme activity (0.5 IU L^−1^). At 1.0 × 10^−6^, the measured initial rate had no significant increase. This is because of the low sensitivity of the sensor at low concentration levels of ACh^+^ ions. At concentrations ≥5.0 × 10^−5^ M, the measured initial rate had a significant increase. The 1.0 × 10^−4^ M ACh^+^ solution was chosen for all subsequent AChE measurements because it revealed a good measurable change in the reaction rate at low activity for the enzyme. As shown in Figure 6, it provided values of 8.9 × 10^−5^ M and 59 mV min^−1^ for *K_m_*, and *V_max_*, respectively. This value of *K_m_* is close to the magnitude obtained previously [35,42].

**Figure 7:**
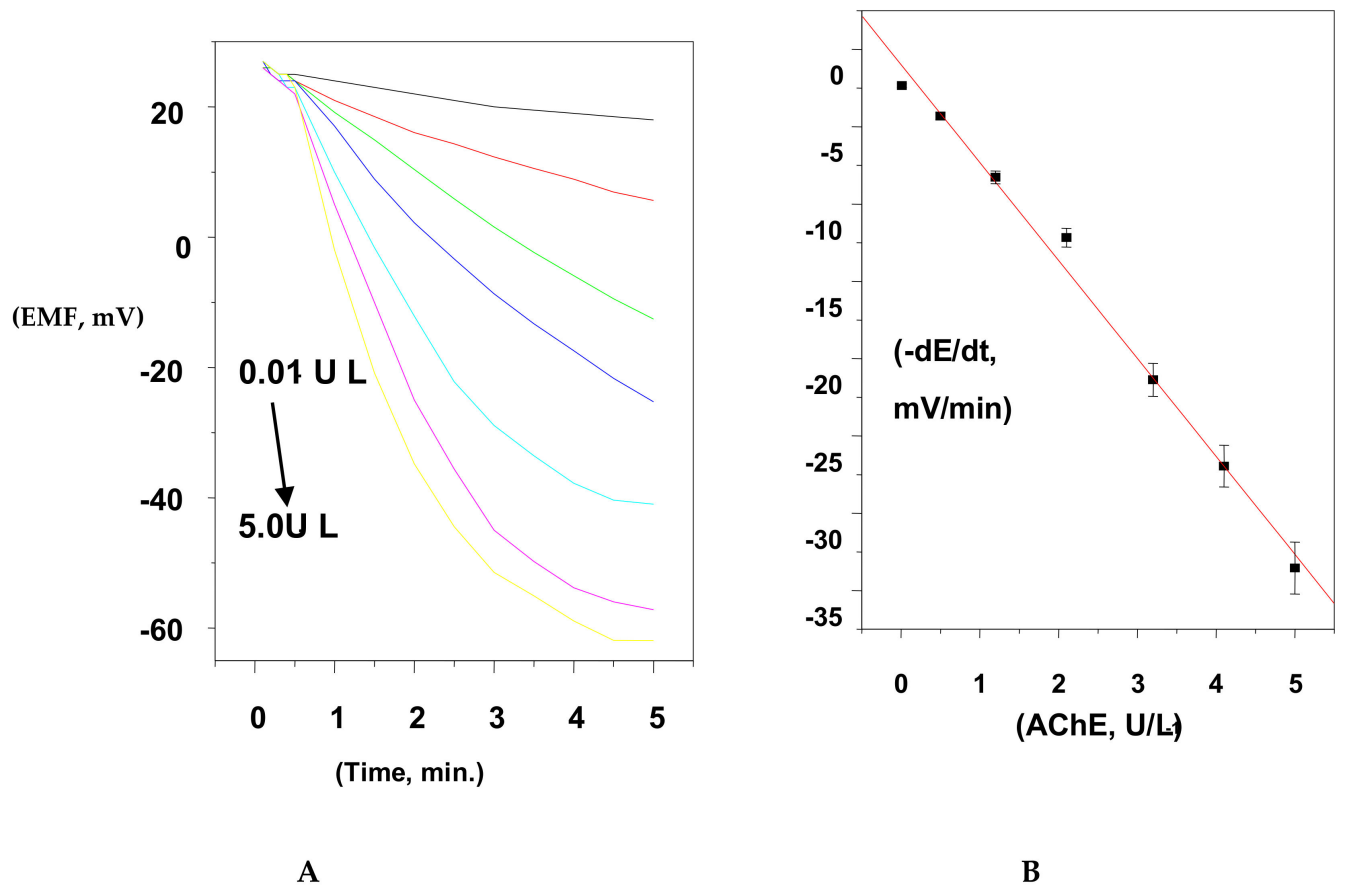
(**A**) Hydrolysis of ACh substrate using the initial rate method at 25 °C; (**B**) the calibration curve constructed for AChE quantification.

For estimation of AChE activity, the 5.0 × 10^−4^ M ACh^+^ concentration was used as a fixed substrate concentration, and the activity of the enzyme was changed from 1.0 × 10^−3^ to 6.0 IU/L. The potential change was recorded versus time, and the initial rate was plotted versus AChE activity. A linear plot (*R*^2^ = 0.9994) was obtained in a concentration range of 5.0 × 10^−3^ to 5.2 IU/L with a detection limit of 3.0 × 10^−3^ IU/L, as shown in Figure 7.

## 3. Materials and Methods

### 3.1. Chemicals and Reagents

All chemicals used in the work were of analytical grade and all solutions were prepared using deionized water (conductivity <0.1 µS cm^−1^). Acetylcholine chloride (ACh), choline chloride (Ch), creatinine (Creat), creatine (Crt), potassium tetrakis (4-chlorophenyl) borate (KTpClPB), sodium tetraphenylborate (NaTPB), 2-nitrophenyloctyl ether (*o*,NPOE), tetradodecylammonium tetrakis (4-chlorophenyl) borate (ETH 500), poly (3,4-ethylenedioxythiophene)/poly-(styrenesulfonate) (PEDOT/PSS), triacetyl-β-cyclodextrin (β-CD), and high-molecular-weight poly(vinyl chloride) (PVC) were purchased from Sigma Chemicals Co. (St. Louis, MO, USA). Tetrahydrofuran (THF) was purchased from Fluka (Ronkonoma, NY, USA). Acetyl cholinesterase (type VI-S) from Electrophorus electricus (electriceel, EC 3.1.1.7, 288 U/mg solid) was obtained from Sigma-Aldrich (Munich, Germany).

A 1.0 × 10^−1^ M stock ACh^+^ solution was prepared by dissolving the salt in 100 mL of 0.05 M phosphate-buffered saline solution (PBS), pH 7. The working standard solutions (1.0 × 10^−2^–1.0 × 10^−8^ M) were prepared daily after stock solution dilution. For the potentiometric selectivity study, a 1.0 × 10^−2^ M interfering ion solution was also prepared using 0.05 M PBS, pH 7.

### 3.2. Apparatus

All EMF measurements were carried out at ambient temperature using a pH/mV meter (Orion, Cambridge, MA, USA, model SA 720) connected with a data logger (Pico Technology Limited, model ADC-16). The flow injection (FI) manifold consisted of an Ismatech peristaltic pump (Ms–REGLO model) with polyethylene tubing (0.71 mm internal diameter) for carrying solutions and an Omnifit injection valve (Omnifit, Cambridge, UK) with a loop sample of 100-μL volume.

For potential stability and capacitance estimation of the solid-contact material used, chronopotentiometry tests were carried out in 10^−3^ M ACh^+^ using a conventional three-electrode system cell. Double-junction Ag/AgCl was used as a reference electrode, and a platinum plate was used as the auxiliary electrode. The applied constant current on the sensors was ±1 nA for 60 s.

### 3.3. ISE Membranes and Electrodes Measurements

**Figure 8:**
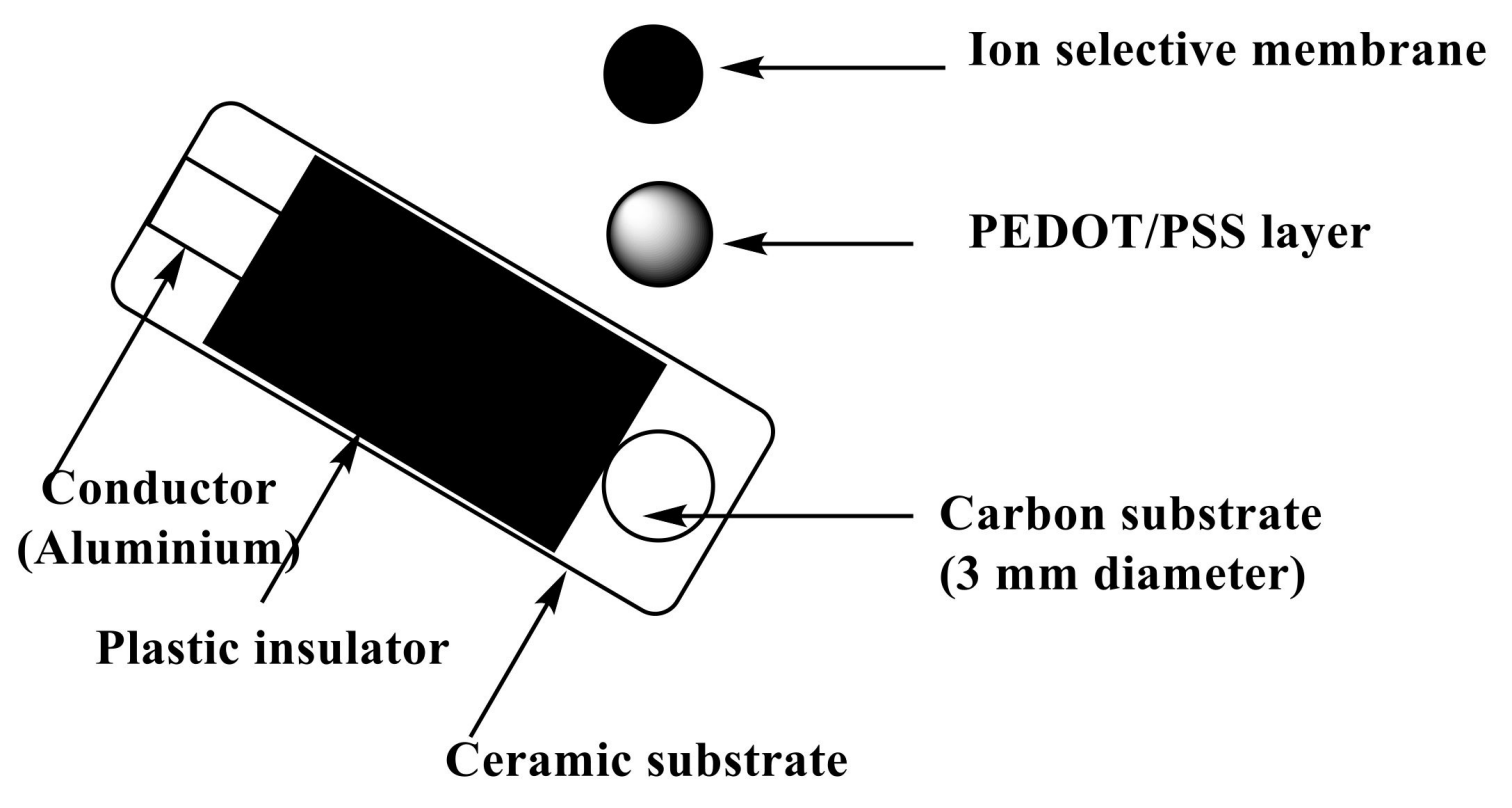
Schematic illustration of fabrication of the potentiometric screen-printed sensor.

To the conductive carbon layer in the screen-printed platform (Figure 8), 10 µL of PEDOT/PSS solution was applied by drop-casting on this orifice. After drying, a layer with a thickness close to 0.25 µm was formed and used as the solid-contact layer. The sensor membrane was prepared by dissolving 100 mg of the total components in 1.0 mL of THF:ionophore (3.0 mg), PVC (32.4 wt.%), ETH 500 (1.5 wt.%), and *o*-NPOE (63.1 wt.%). Then, 20 µL of the membrane cocktail was drop-cast onto the orifice in the screen-printed platform and allowed to dry for 6 h.

The sensors were conditioned by their soaking in 1.0 × 10^−3^ M of ACh^+^ aqueous solution for 2 h. The pH of the test solution was maintained at 7.0 by the addition of different aliquots from standard ACh^+^ solution in 25 mL of 0.01 M PBS solution. The potential of the test solutions was measured at different concentrations of ACh^+^ in the range 1.0 × 10^−8^ to 1 × 10^−2^ M. The EMF was plotted as a function of the logarithm of ACh^+^ concentration.

### 3.4. Acetylcholine Assay in Human Serum

Different human blood samples were collected from different patients and analyzed within 3 h of extraction. The samples were collected in tubes and then mixed with 9 mL of absolute ethyl alcohol; they were left for 10 min before being centrifuged at 4000 rpm. The supernatant liquid was separated from the particulate matter and transferred into a 10-mL measuring flask and completed to the mark by 10^−2^ M phosphate buffer solution of pH 7.2. The cell electrodes were immersed in the buffer solution, and the EMF readings were recorded after electrode potential stabilization. The amount of ACh was calculated using the constructed calibration plot between log [ACh^+^] versus potential readings.

### 3.5. Bioassay of Acetyl Cholinesterase Enzyme (AChE)

To a thermo-stated vessel, a volume of 30.0 mL of the pH 7.0 PBS was transferred, and the cell electrodes were immersed in the solution. After obtaining a stable potential reading for the electrochemical system, 1.0 mL of 10^−2^ M of ACh^+^ working solution was added. When the potential stabilized again, 100-μL aliquots containing 1.0 × 10^−3^–6.0 IU L^−1^ of AChE enzyme were added. From the potential kinetic curve, the potential change with time (Δ*E*/Δ*t*) expressed as initial rate (*t* = 0) was plotted versus enzyme activity. The obtained linear calibration curve was then used for all unknown enzyme activity monitoring. A blank experiment was also carried out under the same conditions but in the absence of the enzyme.

## 4. Conclusions

Herein, we focused on the demonstration of the value of miniaturized screen-printed solid-contact ISEs when facing a complex and relevant determination as an analytical challenge. The work deals with the preparation and characterization of potentiometric acetylcholine-selective membrane sensors using the carbon-based screen-printed ceramic substrate. The PEDOT/PSS showed excellent conductivity when used as the ion-to-electron transducer. The sensors developed were based on the use of TPB ion exchangers and tri acetyl-β-CD ionophore, *o*-nitropheyloctyl ether (*o*-NPOE) as a plasticizer, and PVC as a polymeric matrix. Improved accuracy and precision, good reproducibility, potential stability, rapid response, acceptable selectivity, and high sensitivity were obtained using these sensors. Possible interfacing with automated systems was also offered by these simple and cost-effective potentiometric biosensors. High sample throughputs ranged from ~25–30 samples/h and excellent response characteristics were also obtained using the flow-through system. The activity of acetylcholinesterase (AChE) was also determined using the proposed sensors. The enzyme activity holds 5.0 × 10^−3^–5.2 IU/L.

## Figures and Tables

**Table 1:** General characteristics of some potentiometric sensors for acetylcholine determination.

Ionophore	Slope, (mV Decade^−1^)	Linear Range, (M)	Detection Limit, (M)	pH Range	Interference	Ref.
Acetylcholine dipicrylaminate	54.4	5.0 × 10^−5^–1.0 × 10^−2^	3.0 × 10^−5^	NR	Choline (−1.35); butyrylcholine (−1.02); dopamine (−2.21); tyrosine (−2.39); aminobutyric acid (−2.82); carbachol (−1.43); amphetamine (−1.06); K^+^ (−2.65); NH_4_^+^ (−3.39)	[30]
Cucurbit(6)uril derivative	49.1	1.0 × 10^−6^–1.0 × 10^−3^	9.70 × 10^−7^	7.2	choline (−2.51); NH_4_^+^ (−1.96); NMe_4_^+^ (−1.93); NEt_4_^+^ (−1.93); K^+^ (−1.57); Na^+^ (−1.83); dopamine (−1.51); ascorbic acid (−2.45)	[31]
Dioctyloctadecylamine *N*,*N*-didecyl-aminomethyl -benzene	41.4 52.9	3.0 × 10^−6^–4.5 × 10^−5^ 1.0 × 10^−5^–8.0 × 10^−3^	2.0 × 10^−6^–5.0 × 10^−6^ 5.0 × 10^−6^	8.0 8.0	NR NR	[32]
Tetrakis(*p*-chloro-phenyl) borate	NR	NR	1.0 × 10^−5^	6	NR	[33]
Dibenzo-18-crown-6	NR	NR	1.0 × 10^−5^	6	NR	
Calix[6]arene hexaester	NR	NR	1.7 × 10^−5^	6	NR	
β-Cyclodextrin (β-CD) derivative	55.6	1.00 × 10^−5^–1.0 × 10^−2^	2.70 × 10^−6^	3.0–10	Choline (−2.50); NH_4_^+^ (−3.80); citrate (−2.53); Li^+^ (−3.76); K^+^ (−3.89); caffeine (−2.30)	[34]
Molecularly imprinted polymer (MIP) based on methacrylic acid monomer (MAA) and tetraphenylborate (TPB) additive	55.2	1.00 × 10^−5^–1.0 × 10^−2^	4.5 × 10^−6^	3.0–9.0	Glutamine (−1.52); codeine (−1.37); ephedrine (−1.45); morphine (−1.50); caffeine (−1.5); quinine (−1.57); histidine (−1.60); choline (−1.62); cysteine (−1.70); K^+^ (−2.51); Ca^2+^ (−2.54); Mg^2+^ (−2.82); Ba^2+^ (−2.93)	[35]
Multiwall carbon nanotubes (MWCNTs) and aniline (ANI) in bulk imprinting.	75.9	3.45 × 10^−5^–1.0 × 10^−2^	3.13 × 10^−5^	4.0	Creatinine (−0.04), creatine (+0.82), cysteine (+0.08), glutamine (−0.35), urea (−0.23)	[36]
ACh/TPB/PEDOT/PSS-ISE	56.4 ± 0.6	1.00 × 10^−6^–10 × 10^−3^	2.0 × 10^−7^	3–10	Choline (−3.3), urea (−4.2), hexamine (−4.3), ethylene diamine (−6.2), dimethylamine (−3.2), hydroxylamine (−3.1), methylamine (−3.3), histidine (−4.5), alanine (−4.4), ephedrine (−3.3), codeine (−2.7), morphine (−2.9), K^+^ (−5.3); Ca^2+^ (−6.8); Mg^2+^ (−7.1); Na^+^(−5.3)	This work
β-CD/PEDOT/PSS-ISE	55.3 ± 1.1	2.00 × 10^−6^–1.00 × 10^−3^	3.2 × 10^−7^	4.5–10	Choline (−3.5), urea (−4.5), hexamine (−4.1), ethylene diamine (−6.1), dimethylamine (−4.9), hydroxylamine (−4.8), methylamine (−4.5), histidine (−4.1), alanine (−4.3), ephedrine (−1.2), codeine (−1.0), morphine (−1.1), K^+^ (−5.1); Ca^2+^ (−7.1); Mg^2+^ (−7.3); Na^+^(−6.2).	

NR: Not reported; PEDOT/PSS: poly(3,4-ethylenedioxythiophene)/poly(styrenesulfonate); ISE: ion-selective electrode.

**Table 2:** Potentiometric response characteristics of acetylcholine membrane-based sensors.

Parameter	ACh/TPB/PEDOT/PSS-ISE	β-CD/PEDOT/PSS-ISE
Slope, (mV decade^−1^) *	56.4 ± 0.6	55.3 ± 1.1
Correlation coefficient, (*R*^2^)	0.999	0.998
Linearity range, M *	1.0 × 10^−6^–1 × 10^−3^	2.0 × 10^−6^–1.0 × 10^−3^
Detection limit, M *	2.0 × 10^−7^	3.2 × 10^−7^
Working range, (pH)	3–10	4.5–10
Response time, (s)	<10	<10
Accuracy, (%)	99.2	99.3
Precision, (%)	0.6	0.7

* Average of six measurements. After a rapid 10-fold increase in the concentration of ACh^+^, the time response of the screen-printed sensors to reach steady potential readings was <5 s. The time traces of the two proposed sensors are shown in Figure 1.

**Table 3:** Selectivity values (*K^pot^_ACh,j_*) for acetylcholine solid-contact sensors.

Interferents	log *K^pot^_ACh,j_*	
	ACh/TPB/PEDOT/PSS-ISE	β-CD/PEDOT/PSS-ISE
Choline	−3.3 ± 0.4	−3.5 ± 0.3
Na^+^	−5.3 ± 0.7	−6.2 ± 0.4
K^+^	−5.3 ± 0.3	−5.1 ± 0.1
Ca^2+^	−6.8 ± 0.4	−7.1 ± 0.4
Mg^2+^	−7.1 ± 0.1	−7.3 ± 0.6
Urea	−4.2 ± 0.3	−4.5 ± 0.7
Hexamine	−4.3 ± 0.2	−4.1 ± 0.6
Ethylene diamine	−6.2 ± 0.2	−6.1 ± 0.4
Dimethylamine	−3.2 ± 0.2	−4.9 ± 0.6
Hydroxylamine	−3.1 ± 0.3	−4.8 ± 0.2
Methylamine	−3.3 ± 0.4	−4.5 ± 0.3
Histidine	−4.5 ± 0.3	−4.1 ± 0.4
Alanine	−4.4 ± 0.4	−4.3 ± 0.2
Ephedrine	−3.3 ± 0.4	−1.2 ± 0.3
Codeine	−2.7 ± 0.3	−1.0 ± 0.2
Morphine	−2.9 ± 0.6	−1.1 ± 0.1

**Table 4:** Flow-through response characteristics of acetylcholine membrane-based sensors.

Parameter	ACh/TPB/PEDOT/PSS-ISE	β-CD/PEDOT/PSS-ISE
Slope (mV/decade) *	60.1 ± 1.1	52.7 ± 0.8
Correlation coefficient	0.995	0.996
Detection limit, (M *)	2.5 × 10^−6^	3.9 × 10^−6^
Linear range, (M *)	1.0 × 10^−3^–1.0 × 10^−5^	1.0 × 10^−3^–2.0 × 10^−5^
Optimized flow rate, (mL/min)	4.0	4.0
Life span, (week)	8	8
Sample throughputs, (h)	24–25	29–30

* Average of six measurements.

**Table 5:** Potentiometric determination of ACh in human serum samples using ACh/TPB/PEDOT/PSS-ISE and spectrophotometry.

Sample	Gender	Age	ACh Amount, µM *	
			Potentiometry	Spectrophotometry
1	Male	55–65	0.60 ± 0.03	0.52 ± 0.03
2	Male	35–50	0.48 ± 0.02	0.55 ± 0.04
3	Male	20–30	0.71 ± 0.01	0.57 ± 0.01
4	Female	40–55	0.46 ± 0.02	0.41 ± 0.02
5	Female	20–35	0.53 ± 0.04	0.46 ± 0.05

* Average of five measurements from each individual.
